# Supplementary material for: Persistent homology classification algorithm
Source: PeerJ Comput Sci. 2023 Jan 10;9:e1195. doi: 10.7717/peerj-cs.1195 (PMC10280283; doi:10.7717/peerj-cs.1195)
Supplement: Supplemental Information 11 [file peerj-cs-09-1195-s011.pdf]

APPENDIX

Homology of Simplicial Complexes

Listed here are the basic concepts in algebraic topology which are necessary in understanding of persistent homology. Definitions and theorems are taken mainly from Bubenik (2015), Carlsson (2009), Edelsbrunner and Harer (2008), Ghrist (2008), Pun et al. (2018) Otter et al. (2017) and Zomorodian and Carlsson (2005).

Simplices.

One way of associating an algebraic and combinatorial structure to a topological space is by use of simplicial complexes.

Definition 1 *A k-**simplex**, Δ^k, is the convex hull of $k+1$ points which do not lie in a hyperplane of dimension k or less. It can also be denoted as $[v_0, v_1, v_2, ..., v_k]$, where v_i's are the vertices of Δ^k which has the natural ordering and k is its dimension.*

Definition 2 *A **face** of a k-simplex $[v_0, v_1, v_2, ..., v_k]$ is a simplex $[v_{i_1}, v_{i_2}, ..., v_{i_k}]$ where $i_j \in \{0, 1, 2, ..., k\}$ for each j and $0 \leq i_1 < i_2 < i_3 < ... < i_k \leq k$. If a simplex σ' is a face of a simplex σ, then it is denoted as $\sigma' \subseteq \sigma$. If the dimension of σ' is less than the dimension of σ, then $\sigma' \subset \sigma$.*

A 0-simplex can be represented as a point, a 1-simplex as an edge from one vertex to another vertex, a 2-simplex as a triangular region defined by 3 non-collinear points , a 3-simplex as a tetrahedron together with its interior defined by 4 non-coplanar points, and so on.

Simplicial Complexes.

The k-simplices are regarded as building blocks of simplicial complexes. Simplices can be glued together to form simplicial complexes. A simplicial complex is formally defined as follows.

Definition 3 *Let K^0 be a set of vertices. A **simplicial complex**, K, is a collection of simplices whose vertices are element of K^0, such that if $v \in K^0$ then $[v] \in K$ and if τ and σ are simplices such that $\sigma \in K$ and $\tau \subset \sigma$ then $\tau \in K$. Moreover, the dimension of K is the maximum of the dimensions of its elements.*

Definition 4 *Let K^i denote the set of i-simplices in a simplicial complex K. The **n-skeleton** of K is the union of the sets K^i for all $i \in \{0, 1, 2, ..., n\}$. If σ_1 is a simplex of dimension n_1 and σ_2 is a simplex of dimension n_2, such that $\sigma_1 \subset \sigma_2$, then σ_1 is said to be a face of σ_2 of codimension $n_2 - n_1$.*

Example 1 *Simplicial Complex A*

$$A = \{[a], [b], [c], [d], [e], [a,b], [b,c], [a,c], [d,a], [c,d], [a,b,c]\}$$

Example 2 *Simplicial Complex B*

$$B = \{[f], [g], [h], [f,g], [g,h], [f,h], [f,g,h]\}$$

Simplicial complex A is of dimension 2 since it contains a 2-simplex and it is the element of A with the largest dimension. Similarly, B is of dimension 2.

A simplicial complex can be referred to as an abstract simplicial complex because of its abstract nature. But, one can interpret a finite simplicial complex geometrically as a subset of $\mathbb{R}^n$ for some natural number n. Such subset is called a geometric realization and it is unique up to a canonical piecewise-linear homomorphism (Otter et al., 2017). That is, for a simplicial complex K, there exists a geometric simplicial complex G whose vertices are in one-to-one correspondence with the vertices of K and a subset of vertices in K define a simplex in G if and only if they correspond to the vertices of some simplex of K.

Figure 1 shows the respective geometric realization of simplicial complexes A and B in $\mathbb{R}^2$.

Note that a simplicial complex Δ can also be viewed as a topological space expressed as a quotient of disjoint union of simplices by an equivalence relation that identifies certain faces of certain simplices.

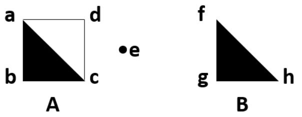

Figure 1. Geometric Realization of A and B

Homology of Simplicial Complexes.

A formal sum of k-simplices is called a k-chain and the free abelian group having a collection of k-simplices as its basis is called a chain group.

Let X be a simplicial complex and $\Delta_k(X)$ be the free abelian group generated by the k-simplices of X. Elements of $\Delta_k(X)$ are called k-simplicial chains. For any $k \in \{1,2,3,...\}$, define the boundary map as the linear map

$$\partial_k : \Delta_k(X) \to \Delta_{k-1}(X),$$
$$\sigma \mapsto \sum_{\tau \subset \sigma, \tau \in \Delta^{n-1}} \tau.$$

The boundary map ∂_k maps each k-simplex to its boundary, which is the sum of its faces of codimension 1. The map ∂_0 is called the zero map. It can be shown that $\partial_n \circ \partial_{n+1} = 0$, that is the boundary of a boundary is always empty. Moreover, the image of ∂_{n+1} is contained in the kernel of ∂_k.

The boundary operators and the chain groups form into a chain complex C_*:

$$\cdots \to \Delta_{k+1} \xrightarrow{\partial_{k+1}} \Delta_k \xrightarrow{\partial_k} \Delta_{k-1} \to \cdots.$$

Definition 5 *For each $n \in \{0,1,2,3,...\}$, the n-th homology of a simplicial complex X, is given as*

$$H_n(X) := Ker(\partial_n)/Im(\partial_{n+1}).$$

Moreover, its dimension

$$\beta_n(X) := dimH_n(X) = dimKer(\partial_n) - dimIm(\partial_{n+1})$$

is called the n-th Betti number of X, or the rank of the n-th homology group of (X). And, elements of $Im(\partial_{n+1})$ are called n-boundaries, and elements of $Ker(\partial_n)$ are called n-cycles.

The n-cycles which are not boundaries represent n-dimensional holes. Thus, the n-th Betti number gives the number of n-holes. Particularly, the $\beta_0(X)$ gives the number of connected components, the $\beta_1(X)$ gives the number of tunnels, the $\beta_2(X)$ gives the number of voids, and so on. Furthermore, if X is a simplicial complex of dimension p, then $H_n(X) = 0$ for each $n > p$. Then there is the following sequence,

$$0 \xrightarrow{\partial_{n+1}} \Delta_n(X) \xrightarrow{\partial_n} \cdots \xrightarrow{\partial_2} \Delta_1(X) \xrightarrow{\partial_1} \Delta_0(X) \xrightarrow{\partial_0} 0.$$

Example 3 *Consider the simplicial complex $A = \{[a],[b],[c],[d],[e],[a,b],[b,c],[a,c],[d,a],[c,d],[a,b,c]\}$ from Example 1 with the geometric realization given in Fig. 2.*

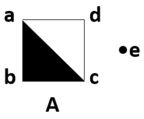

Figure 2. Geometric Realization of A and B

Then there is the following sequence,

$$0 \cdots 0 \xrightarrow{\partial_3} \Delta_2(A) \xrightarrow{\partial_2} \Delta_1(A) \xrightarrow{\partial_1} \Delta_0(A) \xrightarrow{\partial_0} 0,$$

where

$\Delta_0(A) = \mathbb{Z}^5 = span_{\mathbb{Z}}\{[a],[b],[c],[d],[e]\}$,
$\Delta_1(A) = \mathbb{Z}^5 = span_{\mathbb{Z}}\{[a,b],[b,c],[a,c],[c,d],[d,a]\}$,
$\Delta_2(A) = \mathbb{Z} = span_{\mathbb{Z}}\{[a,b,c]\}$, and
$\Delta_k(A) = 0$ for each $k \geq 3$.

Also, for $k = 1,2,3$, the boundary operator ∂_k is defined for k-simplices, respectively as follows,
$\partial_0([x]) = 0$ for each $[x] \in \Delta_0(A)$,
$\partial_1([x,y]) = [y] - [x]$ for each $[x,y] \in \Delta_1(A)$, and
$\partial_2([x,y,z]) = [x,y] + [y,z] - [x,z]$ for each $[x,y,z] \in \Delta_2(A)$.

The homology groups are computed as follows,

$$H_0(A) = \frac{ker\partial_0}{im\partial_1}$$
$$= \frac{span_{\mathbb{Z}}\{[a],[b],[c],[d],[e]\}}{span_{\mathbb{Z}}\{([b]-[a])+([c]-[b]),([c]-[a])+([d]-[c]),[a]-[d]+[c]-[a]\}}$$
$$= \frac{span_{\mathbb{Z}}\{[a],[b],[c],[d],[e]\}}{span_{\mathbb{Z}}\{[a]-[c],[a]-[d],[d]-[c]\}} = \frac{\mathbb{Z}^5}{\mathbb{Z}^3} = \mathbb{Z}^2,$$
$$H_1(A) = \frac{ker\partial_1}{im\partial_2} = \frac{span_{\mathbb{Z}}\{[c,d],[d,a]\}}{span_{\mathbb{Z}}\{[a]+[b]-[c]\}} = \frac{\mathbb{Z}^2}{\mathbb{Z}^1} = \mathbb{Z},$$
$$H_2(A) = \frac{ker\partial_2}{im\partial_3} = 0, \text{ and}$$
$$H_k(A) = 0 \text{ for each } k \geq 3.$$

The Betti numbers are $\beta_0 = 2$, $\beta_1 = 1$, $\beta_2 = 0$, which means that there are 2 connected spaces, 1 hole and 0 voids in A.

For the succeeding sections, simplicial homology will be defined over the field $\mathbb{F}_2$ with 2 elements, where $1 \neq -1$. So instead of defining the chain groups as free abelian groups, we define the chain groups as vector spaces over $\mathbb{F}_2$. However, when computing simplicial homology over $\mathbb{F}_2$, one needs to be careful when defining the boundary maps ∂_k to ensure that $\partial_k \circ \partial_{k+1}$ remains the zero map (Otter et al., 2017). Consequently, the definition is just almost the same, but the resulting homology groups and Betti numbers may vary for different fields. For the purpose of using persistent homology in data science or on Euclidean spaces, it suffices to consider homology with coefficients in the field $\mathbb{F}_2$. Indeed, we will see in the discussion of obtaining topological summaries in the form of barcodes that we will need to compute homology with coefficients in a field, particularly $\mathbb{F}_2$. Furthermore, most of the implementations for the computation of persistent homology in the examples work with $\mathbb{F}_2$.

Computing Persistent Homology of a Point Cloud

Presented here is the general guideline in computing persistent homology of a dataset which follows the pipeline of computing persistent homology as presented in Otter et al. (2017).

Data can be viewed as a collection of points in a metric space. This finite metric space is also called a point cloud. Points in the dataset or point cloud are thickened gradually and this gradual evolution of the point clouds' shape and its topological properties are now the point of interest for topologist and data scientists.

Let X_0 be a finite subset of a Euclidean space R^n. Then, X_0 is an example of a point cloud. These points which can be viewed as vertices which will serve as building blocks of complexes.

Let ε be a non-negative real number which serves as parameter to thicken X_0 and X_ε be the thickened point cloud. Homology of a given data pertains to topological invariant properties of X_ε which can be computed algebraically. That is, for each nonnegative integer i, there is a corresponding vector space or a homology group $H_i(X_\varepsilon)$. The dimension of the first 3 homology groups gives the number of connected components, the number of tunnels or holes and the numbers of voids, respectively. This algebraic structures are said to be robust or homotopy invariant. That is, a primary space's topological invariant features does not change when the space undergo bending, stretching or other deformations. Furthermore, computing homology of a finite simplicial complex can be easily done with the aid of linear algebra.

Computing the homology of an arbitrary topological space is not as straight forward as that of computing the homology of a finite simplicial complex. Firstly, one has to find a simplicial complex whose homology approximates the homology of the arbitrary space. There are various ways of doing this, and the most natural methods are with the use of Čech complexes and Vietoris-Rips complexes. There are alternative complexes like Delaunay complex, Alpha complex and Witness complex.

Let the parameter ε be a non-negative real number and X be a set of points in the Euclidean space $\mathbb{E}^k$ and $\mathscr{U} = \{\mathrm{U}_i\}_{i\in I}$ be a cover of X. The k-simplices of the Čech complex are the non-empty intersections of $k+1$ sets in $\mathscr{U}$.

Definition 6 *Let $\mathscr{U} = \{\mathrm{U}_i\}_{i\in I}$ be the non-empty collection of sets. The nerve of $\mathscr{U}$ is the simplicial complex with the vertices given by I and the k-simplices given by $\{i_0, i_1, i_2, ..., , i_k\}$ if and only if $\bigcap_{j\in\{0,1,...,k\}} \mathrm{U}_{i_j} \neq \emptyset$.*

Theorem 1 (Nerve Theorem) *The geometric realization of the nerve of $\mathscr{U}$ is homotopy equivalent to the union of sets in $\mathscr{U}$.*

Definition 7 *The Čech complex with parameter ε of X is given as*

$$\check{C}_\varepsilon(X) := \{\sigma \in X | \bigcap_{x\in\sigma} B(x,\varepsilon) \neq \emptyset\}$$

where $B(x,\varepsilon)$ is a closed ball of radius ε centered at x.

If the cover of the sets in X is sufficiently 'nice,' then the Nerve Theorem guarantees that the nerve of the cover and the space X have the same homology (Edelsbrunner and Harer, 2010). However, finding the Čech complex is computationally expensive as it involves investigating a very large number of intersections. Furthermore, the Čech complex may have a higher dimension than the underlying space. This is the reason why choosing Vietoris-Rips (VR) complex,an approximation of the Čech complex, can be more attractive.

Definition 8 *Let (X,d) be a metric space, S be a subspace of X with the induced metric. The Vietoris-Rips complex with parameter ε, denoted by $\mathscr{R}_\varepsilon(X)$, is the set of all $\sigma \subset X$, such that the largest Euclidean distance between any of its points is at most 2ε. That is, given $S \subset X$,*

$$\mathscr{R}_\varepsilon(S) = \{\sigma \subseteq S | d(x,y) \leq 2\varepsilon \, \textit{for all} \, x,y \in \sigma\}.$$

Both the Vietoris-Rips complex and the Čech complex are abstract simplicial complexes which may be defined at various parameters ε, but only Čech complex preserves the homotopy information of the topological spaces formed by the ε-balls.

Moreover, given S is a subset of a Euclidean space, the Vietoris-Rips complex approximates the Čech complex in such a way that $\check{C}_\varepsilon(S) \subseteq VR_\varepsilon(S) \subseteq \check{C}_{\sqrt{2}\varepsilon}(S)$.

The construction of a VR complex can be made easier with the use of clique complexes, also known as the flag complexes. In topology, recall that a graph is complete if any two vertices in the graph is connected by an edge and the set of vertices which form a complete graph is called a clique. A k-clique complex is formed from a clique of $k+1$ vertices. Since subsets of a clique is also a clique, then a clique complex is also a simplicial complex.

Now, for easier construction a VR complex of S, form the ε-neighborhood of S which is composed of vertices in S and edges $(i,j) \in S \times S$ where $i \neq j$ and $d(i,j) \leq 2\varepsilon$. Afterwards, compute the clique complex of the ε-neighborhood graph. This construction is easier for the reason that one only needs to check for pairwise distances to construct a clique complex. Although this technique is computationally less expensive as that of computing the Čech complex, the VR complex may have the same worst-case complexity as that of the Čech complex. That is, in the worst case, a VR complex may have $2^{|S|}-1$ simplices and dimension $|S|-1$. Moreover, one can opt to just compute the VR complex up to some dimension $k \ll |S|-1$. Otter et al. (2017) used $k=2$ and $k=3$ in their simulations.

Example 4 *Consider $K = \{(0,0),(1,0),(0,0.5),(1,1)\} \subseteq \mathbb{R}^2$ and the filtration parameters values $\varepsilon_1 = 0$, $\varepsilon_2 = 0.2$, $\varepsilon_3 = 0.4$, $\varepsilon_4 = 0.6$ and $\varepsilon_5 = 0.8$.The first column of Fig. 3 shows the filtration of K using Čech complexes, with $C_1 \subseteq C_2 \subseteq ... \subseteq C_5$. The second column of Fig. 3 show the filtration of K using Vietoris-Rips complexes, with $VR_1 \subseteq VR_2 \subseteq ... \subseteq VR_5$.*

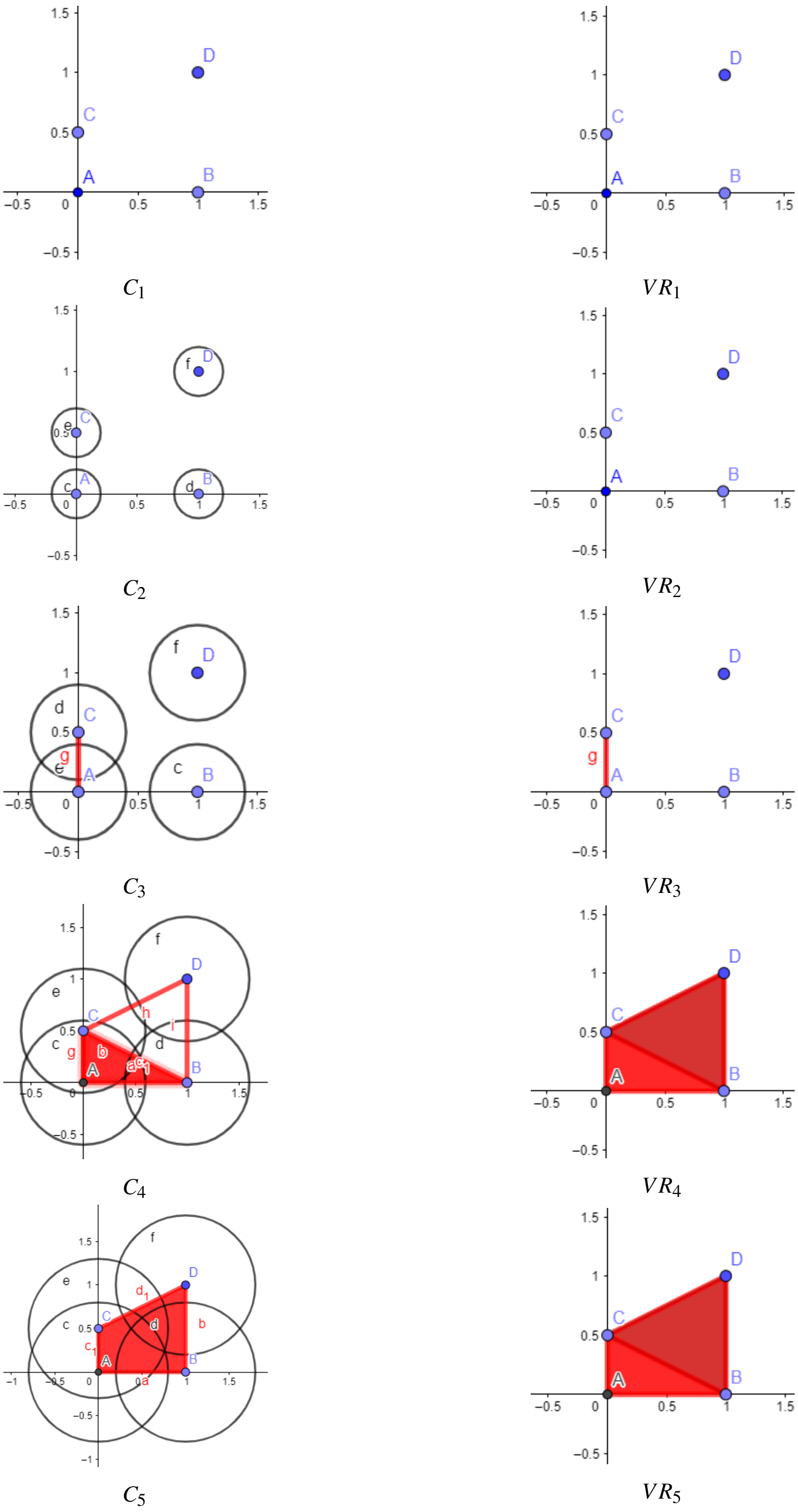

Figure 3. Filtration of K using Čech and Vietoris-Rips Complexes

140 In this particular example, the Čech complexes and the Vietories-Rips complexes differ only at the
141 4-th filtration index.

Given a finite metric space S, say a set of experimental dataset, it is assumed that the data is a sample from some underlying topological space. The goal in computing the persistent homology of this data is to recover the properties of such underlying topological space while maintaining the robustness of the data. Given a subset S of a Euclidean space, one can consider S_ε, a simplicial complex at different values of ε. As the value of ε increases, simplices are added to the complexes and sequence of nested simplicial complexes is formed. This is the part where the homology of the simplicial complexes changes as the parameter ε changes. The goal now in is to determine which topological features persist across the changes in the ε values. Thus, the name persistent homology.

Definition 9 *Let K be a finite simplicial complex and $K_1 \subseteq K_2 \subseteq ... \subseteq K_r = K$ be a finite sequence of nested subcomplexes of K. K is called a filtered simplicial complex and the sequence $\{K_1, K_2, ...\}$ is called the filtration of K.*

Homology of each of the subcomplexes can be computed. For each p, the inclusion maps $K_i \to K_j$ induce $\mathbb{F}_2$-linear maps $\partial_i^j : H_p(K_i) \to H_p(K_j)$ for all $i, j \in \{1, 2, ..., r\}$ with $i \leq j$. It follows from functoriality that $\partial_k^j \circ \partial_i^k = \partial_i^j$ for all $i \leq k \leq j$.

Definition 10 *Let K_s be a subcomplex in the filtration of the simplicial complex K, or K_s be the filtered complex at time s, and $Z_k^s = Ker\partial_k^s$ and $B_k^s = Im\partial_{k+1}^s$ be the k-th cycle group and boundary group of K_s, respectively. The k-th homology group of K_s is $H_k^s = Z_k^s / B_k^s = Ker(\partial_k^s)/Im(\partial_{k+1}^s)$.*

Definition 11 *For $p \in \{0, 1, 2, ...\}$, the p-persistent k-th homology group of K given a subcomplex K_s is K_s is*

$$H_k^{s,p}(K, K_s) = H_k^{s,p}(K) = Z_k^s / (B_k^{s+p} \bigcap Z_k^s) = \frac{Ker(\partial_k^s)}{Im(\partial_{k+1}^{s+p}) \bigcup Ker(\partial_k^s)}.$$

The p-th persistent k-th Betti number $\beta_k^{s,p}$ of K_s is the rank of $H_k^{s,p}(K)$. Note that the zero-persistent homology groups of K_s are the same as the actual homology groups of K_s.

The results of computing the persistent homology of a filtered simplicial complex are normally given in terms of persistent pairs consisting of birth times and death times. And, these are normally visualized in various ways. The most common way of visualizing results of computing persistent homology is by use of persistence barcodes. These are representations of the recorded birth times and death times of the topological invariant properties or generators. Birth times and death times refer to the filtration values (ε values or filtration time) at which the generators appeared and vanished, respectively. The name persistent barcode was first used in Zomorodian and Carlsson (2005) and discussed in more details in Ghrist (2008). But, the standard algorithm for transforming persistent homology into barcodes is presented here as seen in Otter et al. (2017), as their presentation is clear, concise and beginner friendly.

Given a filtered simplicial complex K, with filtration $K_0 \subseteq K_1 \subseteq K_2 \subseteq ... \subseteq K_r$. The persistent pair may be given by the pair (b, d), where $b \in \{0, 1, 2, ..., r\}$ is the birth time, $d \in (\{0, 1, 2, ..., r\} \cup \infty)$ is the death time, $b \leq d$ and $d - b$ is the length or lifespan of the homology. If $d < \infty$ then the generator vanishes at filtration time d and if $d = \infty$ then the homology persists on all the succeeding filtration steps.

The persistent barcode for the filtered simplicial complex K can be created using the following steps. First, K must be associated to boundary matrix whose entries represents faces of the simplexes. It is assume that each of the simplexes of the nested sequence of complexes follow a total ordering such that a face of a simplex precedes the simplex and a simplex in the i-th complex K_i precedes the simplices in K_j for $j > i$, which are not in K_i. Let n be the total number of simplices in the complex, and $\sigma_1, \sigma_2, ..., \sigma_n$ be the simplices. The square matrix B, of dimension $n \times n$, is constructed by assigning a value 1 in $B(i, j)$ if the simplex σ_i is a face of simplex σ_j of codimension 1 and a value 0 otherwise. That is, the boundary matrix B is defined by

$$B(i, j) = \begin{cases} 1, & \text{if } \sigma_i \subset \sigma_j \text{ and } dim(\sigma_j) - dim(\sigma_i) = 1 \\ 0, & \text{otherwise.} \end{cases}$$

After constructing the boundary matrix B, it has to be reduced using the *standard algorithm*, sometimes called column algorithm, for the computation of PH. The standard algorithm used for computing PH was first introduced in Edelsbrunner et al. (2002). For each $j \in \{1, 2, ..., n\}$, define $low(j)$ to be the largest

value i such that $B(i,j)$ is different from 0. If column j only contains 0 entries, then the value of $low(j)$ is undefined. Boundary matrix B is reduced if the map low is injective on its domain of definition. In the worst case, the complexity of the standard algorithm is cubic in the number of simplices (Otter et al., 2017).

Finally, the reduced boundary matrix can now be encoded into a barcode. This is done by pairing the simplices in the following manner:

- If $low(j) = i$, then the simplex σ_j is paired with σ_i, and the appearance of σ_i in the filtration causes the birth of a feature that dies with the entrance of σ_j.
- If $low(j)$ is undefined, then the appearance of the simplex σ_j in the filtration causes the birth of a feature. If there exists k such that $low(k) = j$, then σ_j is paired with the simplex σ_k, whose appearance in the filtration causes the death of the feature. If no such k exists, then σ_j is unpaired.

A pair (σ_i, σ_j) gives the half-open interval $[dg(\sigma_i), dg(\sigma_j))$ in the barcode, where for a simplex $\sigma \in K$ we define $dg(\sigma)$ to be the smallest number l such that $\sigma \in K_l$. An unpaired simplex σ_k gives the infinite interval $[dg(\sigma_i), \infty)$.

Definition 12 *Let K be a set of points in the Euclidean space $\mathbb{R}^d$. Fix $m \in \mathbb{N}$ be the number of simplicial complexes in the filtration of K. Suppose $K^0 \subseteq K^1 \subseteq K^2 \subseteq \cdots \subseteq K^m$ is a filtration of K with respect to parameter values ε_i's such that $0 = \varepsilon_0 < \varepsilon_1 < \varepsilon_2 < \cdots < \varepsilon_m$. Let $n \in \mathbb{N}$ be the number of simplices σ_j's in K^m. For each $k \in \{0,1,2,...,d\}$, there is a barcode B_k which is a collection of half-open intervals $[\sigma_{j,1}, \sigma_{j,2})$, which are pairs of birth time and death time of a generator in $H_k(K)$. Then, for each k, $k = 0,1,2,...d$ the number of infinite intervals in B_k gives the k-th Betti number or the rank of $H_k(K^m)$. And, the persistent homology of K based on the filtration $K^0 \subseteq K^1 \subseteq K^2 \subseteq \cdots \subseteq K^m$ is the homology of K^m.*

Example 5 *Consider a set of points $K = \{(0,0),(0,1),(.5,1.5),(1,1),(1,0)\}$ and filtration values $\varepsilon_1 = 0.2, \varepsilon_2 = 0.4, \varepsilon_3 = 0.6, \varepsilon_4 = 0.8$, and $\varepsilon_5 = 1.0$.*

A filtration of K using the Čech complexes, $\emptyset = K^0 \subseteq K^1 \subseteq K^2 \subseteq K^3 \subseteq K^4 \subseteq K^5$, is given in Fig. 4.

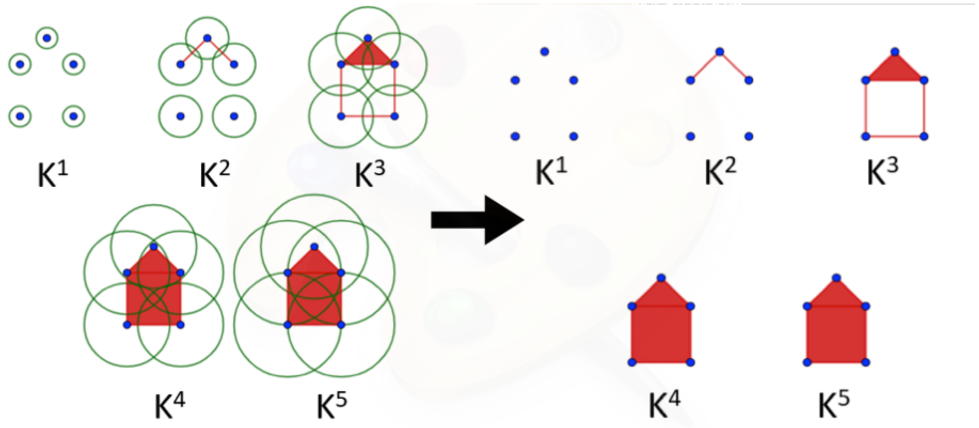

Figure 4. A Filtration of K

After implementing the standard algorithm, its barcodes for degree 0 and degree 1 are shown in Fig. 5 and Fig. 6, respectively.

It can be concluded from the barcodes that the respective Betti numbers are $\beta_0 = 1$ and $\beta_1 = 0$. Based on the given filtration parameter values, the persistent homology of K can be described as a connected space with no hole.

Persistence barcodes are then analyzed by studying properties of metric spaces whose elements are persistence diagrams. A persistence diagram is another form of visualizing results of PH computations. It gives similar information that a barcode provides. Also, distance functions were defined on a space of persistence diagrams.

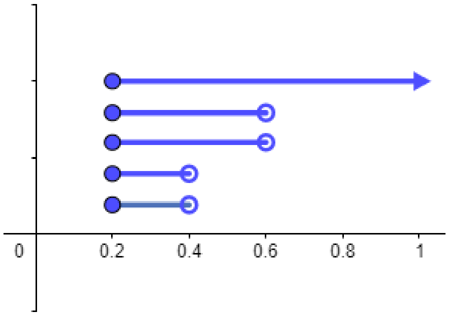

Figure 5. Barcode for degree 0

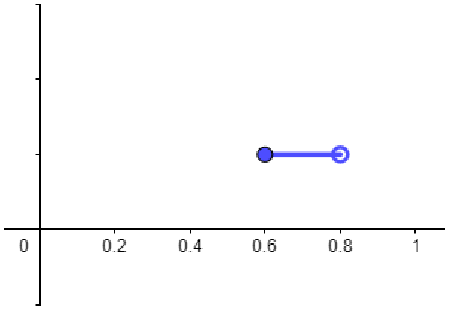

Figure 6. Barcode for degree 1

Recall that the n-th Betti number of a topological space X is denoted by β_n, which is equal to the rank of the n-th homology group H_n. Moreover, if K is a simplicial complex and $\{K_r\}_{r \in J}$ for some indexing is the the filtration of K, the p-th persistent k-th Betti number $\beta_k^{s,p}$ of K_s is the rank of $H_k^{s,p}(K)$. From the persistent Betti numbers, there is a set of multiplicities $\mu_n^{i,j} > i$ such that

$$p = j - i,\ \mu_n^{i,j} = \beta_n^{i,p} - \beta_n^{i-1,p} - \beta_n^{i,p+1} + \beta_n^{i-1,p+1}$$

The multiplicity $\mu_n^{i,j}$ is the number of features in the n-th homology group that appears at filtration i and vanishes at filtration time j.

Definition 13 (Persistence Diagram) *Let $\{K_r\}$ be the filtration of a simplicial complex K. The n-th persistence diagram of K with the filtration $\{K_r\}$, denoted by $PD_n(\{K_r\})$ is a subset of $\bar{\mathbb{R}}^2$, where $\bar{\mathbb{R}}^2 = (\mathbb{R} \cup \{\pm\infty\}) \times (\mathbb{R} \cup \{\pm\infty\})$, with each point (i, j) has a multiplicity of $\mu_n^{i,j}$ and all points in the diagonal where $i = j$ have infinite multiplicity.*

Discussion of the robustness and stability of persistence diagram requires the notion of distance. Given two persistence diagrams, say X and Y, the definition of distance between X and Y is given as follows.

Definition 14 *Let $p \in [1, \infty]$. The p-th Wasserstein distance between X and Y is defined as*

$$W_p[d](X,Y) := \inf_{\phi: X \to Y} \left[\sum_{x \in X} d[x, \phi(x)]^p \right]^{1/p}$$

for $p \in [1, \infty)$ and as

$$W_\infty[d](X,Y) := \inf_{\phi: X \to Y} \sup_{x \in X} d[x, \phi(x)]$$

for $p = \infty$, where d is a metric on $\bar{\mathbb{R}}^2$ and ϕ ranges over all bijections from X to Y.

Normally, d is taken to be L_q where $q \in [1, \infty]$ and the most commonly used distance function is the Bottleneck distance $W_\infty[L_\infty]$.

REFERENCES

Bubenik, P. (2015). Statistical topological data analysis using persistence landscapes. *Journal of Machine Learning Research*, 16.

Carlsson, G. (2009). Topology and data. *Bull Am Math Soc*, 46.

Edelsbrunner, Letscher, and Zomorodian (2002). Topological persistence and simplification. *Discrete & Computational Geometry*, 28(4):511–533.

Edelsbrunner, H. and Harer, J. (2008). Persistent homology — a survey.

Edelsbrunner, H. and Harer, J. (2010). *Computational topology: an introduction*. Am. Math. Soc., Providence.

Ghrist, R. (2008). Barcodes: the persistent topology of data. *Bull Am Math Soc*, 45.

Otter, N., Porter, M. A., Tillmann, U., Grindrod, P., and Harrington, H. A. (2017). A roadmap for the computation of persistent homology. *EPJ Data Science*, 6(1):17.

Pun, C. S., Xia, K., and Lee, S. X. (2018). Persistent-homology-based machine learning and its applications – a survey.
Zomorodian, A. and Carlsson, G. (2005). Computing persistent homology. *Discrete & Computational Geometry*, 33(2):249–274.
